# Electrosmog and autoimmune disease

**DOI:** 10.1007/s12026-016-8825-7

**Published:** 2016-07-13

**Authors:** Trevor G. Marshall, Trudy J. Rumann Heil

**Affiliations:** 1 Autoimmunity Research Foundation, Thousand Oaks, CA USA; 2 NP-Private Practice Associates, Scottsdale, AZ USA

**Keywords:** Electrosmog, VDR, Autoimmune disease, PPPM, WiFi, Electromagnetic hypersensitivity

## Abstract

Studies in mice have shown that environmental electromagnetic waves tend to suppress the murine immune system with a potency similar to NSAIDs, yet the nature of any Electrosmog effects upon humans remains controversial. Previously, we reported how the human Vitamin-D receptor (VDR) and its ligand, 1,25-dihydroxyvitamin-D (1,25-D), are associated with many chronic inflammatory and autoimmune diseases. We have shown how olmesartan, a drug marketed for mild hypertension, acts as a high-affinity partial agonist for the VDR, and that it seems to reverse disease activity resulting from VDR dysfunction. We here report that structural instability of the activated VDR becomes apparent when observing hydrogen bond behavior with molecular dynamics, revealing that the VDR pathway exhibits a susceptibility to Electrosmog. Further, we note that characteristic modes of instability lie in the microwave frequency range, which is currently populated by cellphone and WiFi communication signals, and that the susceptibility is ligand dependent. A case series of 64 patient-reported outcomes subsequent to use of a silver-threaded cap designed to protect the brain and brain stem from microwave Electrosmog resulted in 90 % reporting “definite” or “strong” changes in their disease symptoms. This is much higher than the 3–5 % rate reported for electromagnetic hypersensitivity in a healthy population and suggests that effective control of environmental Electrosmog immunomodulation may soon become necessary for successful therapy of autoimmune disease.

**Electronic supplementary material:**

The online version of this article (doi:10.1007/s12026-016-8825-7) contains supplementary material, which is available to authorized users.

## Introduction

“Electrosmog” describes the electromagnetic waves surrounding us in our environment. According to NASA [1]:

“As you sit watching TV, not only are there visible light waves from the TV striking your eyes, but also radio waves, transmitting from a nearby station, and microwaves carrying cellphone calls and text messages, and waves from your neighbor’s WiFi, and GPS units in the cars driving by. There is a chaos of waves from all across the spectrum passing through your room right now.”

Every year, the quantity and nature of radio and microwaves contained in this Electrosmog increases. However, research into whether they might interact with human biology, and exactly how they might interact, is a field clouded by the jargon and complexity of each technology and hampered by inadequate experimental guidelines.

The only known natural source of microwave electromagnetic radiation is the negligibly weak cosmic radiation from space, although significant sources of natural radiation have existed at lower radio frequencies due to atmospheric phenomena such as the aurora borealis and thunderstorms. Until the 1950s, Electrosmog frequencies remained out of the microwave region, but by the 1960s television channels began microwave transmissions. Cellular phone technologies emerged during the 1980s; WiFi during the 1990s. Both extensively use microwave frequency bands. The recent release of WiGig and anti-collision vehicle radars in the 60 GHz region embody a 1000-fold increase in frequency, and photon energy, over the exposures mankind experienced up until the 1950s.

It is generally accepted that exposure to low-energy radio waves does not produce any sign of harm. However, low-level exposures to ionizing radiation, for example the exposures caused by nuclear energy accidents, do indeed affect human biology. It may take years of accumulated exposure before the subsequent symptoms become apparent.

Both ionizing X-rays and non-ionizing microwaves are forms of electromagnetic radiation. The energy in X-rays, however, is much higher, usually above a thousand electron volts (1 keV), while the energy in each microwave photon is usually just a few micro-electron volts (μeV) [1].

A primary effect of low-dose ionizing radiation (from radon and X-rays) is suppression of our body’s immune defenses [2, 3], something which often does not become apparent until the body catastrophically fails to overcome an acute challenge. The emerging use of radon exposure to mitigate rheumatoid arthritis symptoms in humans [4] is an interesting exploitation of radiation’s immunosuppressive properties.

With low-level non-ionizing electromagnetic radiation, Lushnikov [5] found a suppressed immune response in mice. Subsequently, Gapeev (*aka* Gapeyev) [6, 7] showed that the effect on mice of low-intensity non-ionizing electromagnetic waves was roughly equivalent to effect of the NSAID diclofenac. Most recently, some suppression of inflammation was reported in lizards which had been exposed to pulsed DECT radiation simulating the cordless phones used in many homes [8].

### Proteins are continually in motion, responsive to electromagnetic waves

We have previously reported [9–13] that the drug olmesartan could be retargeted to produce immunostimulation in patients with autoimmune disease. During that research, we used the emerging field of molecular dynamics (MD) to analyze the actions of both the drug olmesartan and the native ligand, 1,25-dihydroxyvitamin-D (1,25-D) on the VDR [14]. Molecular dynamics is computationally intensive, as interactions between each atom in the VDR protein, its activating ligand, and the surrounding water are calculated incrementally as a function of time. We found that hydrogen bond exchange within the VDR exhibited structural resonances at frequencies typically found in modern Electrosmog.

Turton et al. [15] in *Nature Communications* 2013 used MD to study the interaction between lysozyme and its ligand triacetylchitotriose at much higher frequencies than Electrosmog. They were then able to confirm that the lysozyme complex was indeed underdamped by using femtosecond optical Kerr Effect spectroscopy. They concluded that the lysozyme complex was marginally unstable, and non-ionizing terahertz electromagnetic radiation is likely to alter “proper biological function.”^1^

We used MD software to create a movie which allowed us to easily visualize the relative motion of each atom in the VDR as a function of time. The MD output comprises a very large number of incremental combinations of protein and ligand, which can be displayed as frames of a movie film. This allows the relative motions of each atom in the interaction to be studied.

**Fig. 1:**
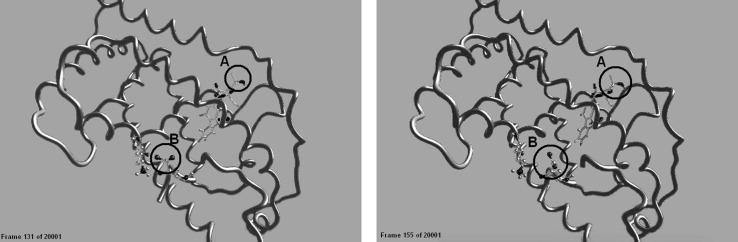
Frames 131 and 155 from a movie showing activation of the VDR by olmesartan, obtained using Gromacs for molecular dynamics emulation. Most of the 239 amino acid residues making up the PDB:1DB1 VDR are shown in helical representation, with the atoms of GLU420 and LYS264 highlighted in “ball and stick” notation. The ligand olmesartan can be seen within *circle* “A” and GLU420 within “B”

Two frames from a movie of a VDR molecule being activated by olmesartan are shown in Fig. 1 (the movie is available as “olmesartan.MP4” in the “Supplementary Information” file). These frames, separated by a time interval of 900 femtoseconds, show the VDR helical “backbone” and the position of several key atoms. The circular area labeled as “B” highlights the carboxyl group of glutamine at position GLU420 in PDB:1DB1, a crystal structure model of the VDR [14].

### How Electrosmog interacts with human metabolism

There is no need to go into detail to understand the action of Electrosmog on human proteins. All one needs to notice is that, in Fig. 1, the two oxygen atoms of the carboxyl group at “B” have spun by 90° in the time between the two frames. Although all the atoms of the VDR are constantly in motion, these two oxygens are key because they are involved in forming hydrogen bonds with the DRIP205 coactivator. When the VDR is not activated, this carboxyl group binds with the lysine to its left (LYS264) and cannot position the coactivator where it needs to be for proper gene transcription. Activation forces these residues apart so they can bind with the coactivator. The shape of the whole VDR molecule changes as it is activated by the drug.

Whenever an electromagnetic field is present, a Lorentz Force [18] will act upon any charged atom in motion, such as these moving oxygen atoms, a force which could either boost or hinder the proper activation of the VDR molecule. Whether activation is assisted or blocked depends on the frequency content of the molecular interactions, and that of the impinging electromagnetic waves.

So why do not human beings suffer immediate symptoms when exposed to Electrosmog? Recall the time interval between the two frames in Fig. 1—900 femtoseconds—corresponding to a wave frequency near 1 terahertz (THz). Very few waves oscillating at that frequency are able to reach the molecules of a human body, and none of them are currently present in Electrosmog. Consequently, Electrosmog does not yet directly affect motions of the individual atoms.

However, at least in the case of the VDR activation, the bulk of the molecule changes shape with characteristic frequencies already found in today’s Electrosmog. Groups of hundreds of atoms which form the helical “backbone” of the VDR do shift together at the lower frequencies present in Electrosmog.

**Fig. 2:**
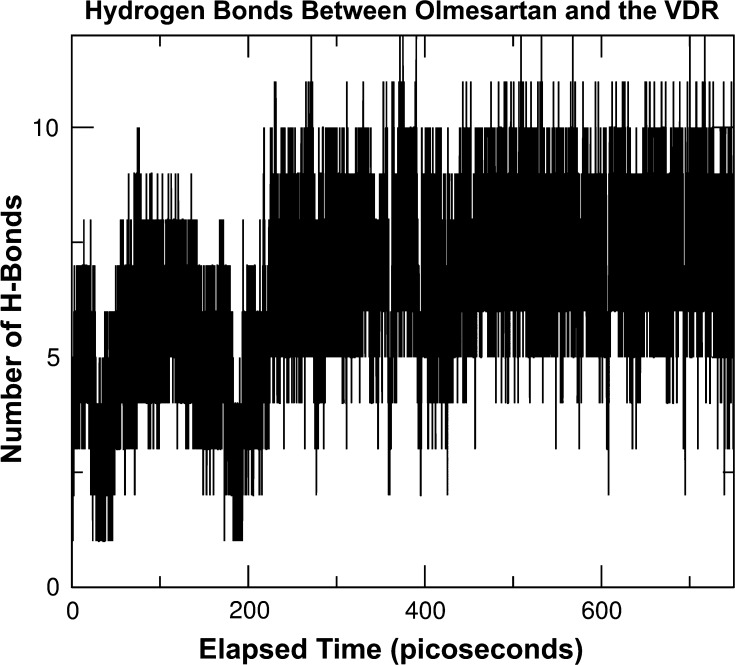
A plot (from the Gromacs g_hbond software) of the instantaneous number of hydrogen bonds formed between olmesartan and the VDR sampled every 37.5 femtoseconds during the first 750 ps of VDR activation

The number of hydrogen bonds formed between olmesartan and the VDR over time shows many periods of marginally stable activation, as can be seen in Fig. 2. Despite an initial 170 ps sinusoidal instability, the number of hydrogen bonds builds to a stable range within 300 ps of the olmesartan getting to the binding pocket. However, even this “stable” region beyond 300 ps shows considerable fluctuation, with a noticeable tendency to oscillations having the same characteristic 170 ps period. An FFT of the data^2^ confirmed a primary response peak at a frequency just below 6 GHz (which corresponds to the 170 ps interval). WiFi routers operate in this frequency range, and these routers already comprise a significant proportion of indoor Electrosmog.

### The VDR is even more susceptible when bound with its natural ligand

**Fig. 3:**
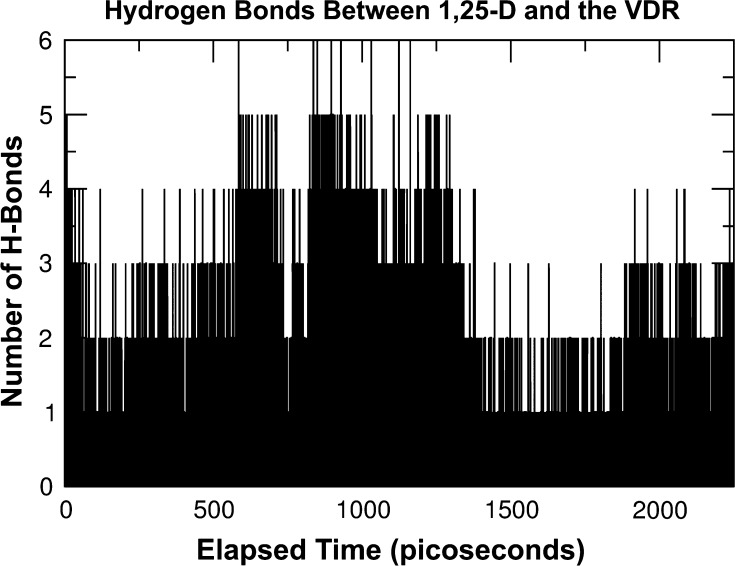
A plot (from the Gromacs g_hbond software) of the instantaneous number of hydrogen bonds formed between 1,25-D and the VDR sampled every 37.5 femtoseconds during the first 2250 ps of VDR activation

The primary natural ligand for the VDR is 1,25 dihydroxyvitamin-D, a ligand with fewer oxygen atoms than olmesartan. The hydrogen bond count for this 1,25-D and VDR combination is therefore lower. Consequently, the VDR “backbone” is less rigid than when olmesartan is used as the ligand. Figure 3 shows 2250 ps of activity, three times as long a time frame as is shown in Fig. 2.

**Fig. 4:**
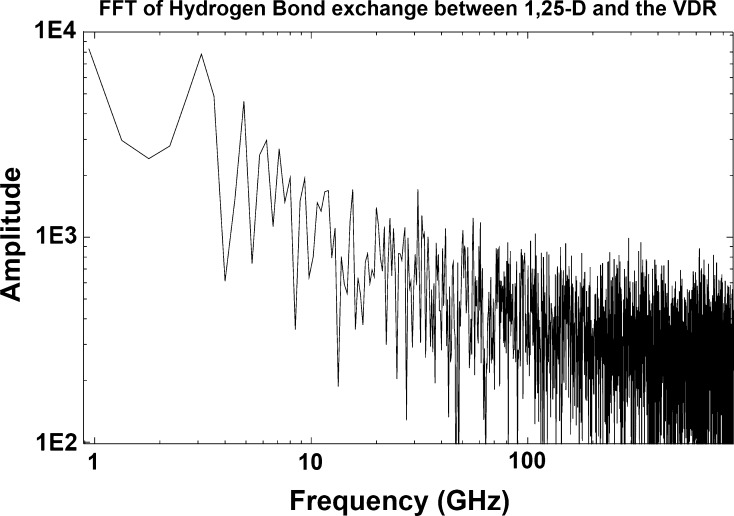
A fast Fourier transform of the hydrogen bond data from Fig. 2

The modes of functional resonance in the natural ligand/VDR combination are slower, and the FFT (Fig. 4) confirms multiple hydrogen bond exchange rate peaks in the 3, 5 and 6 GHz, frequency bands, close to those typically found in Electrosmog from WiFi and 4G-LTE cellular communication devices.

### Electromagnetic waves in Electrosmog exert sufficient force to affect biological function

The force exerted on a moving charge by an electromagnetic wave is dependent on the charge’s velocity, the wave’s frequency and the wave’s amplitude [18]. With environmental Electrosmog, the amplitude is an uncontrolled variable, and amplitudes can easily exceed −16 dBm^3^ (1 V/m at 1 GHz) when close to cell phones, cell towers and WiFi access points.

There are many studies which document biological effects at these higher levels, and the 2012 “BioInitiative” consensus [19] collated and summarized many of them. However, very few studies tried to define the lowest level at which electromagnetic waves might start to affect biology. Bise [20] reported in 1978 that human EEG was changed by wave amplitudes as low as −100 dBm, with −60 dBm giving multiple subjects immediate frontal headache. Sadly, such levels are impossible to replicate in 2016 without the use of a Faraday cage, as the Electrosmog background levels in our cities rarely fall below −50 dBm (100,000 times stronger than the −100 dBm signals used by Bise).

While investigating the report of Gapeev [21] that the near-field zone of an antenna seemed more biologically active than the far-field zone, we received reports that 27.12 MHz signals from our near-field (capacitive wave) antenna, similar in design to Figure 9 of Sacco and Tomilin [22], could be sensed by patients, but not by healthy individuals. This occurred at levels around −90 dBm, levels below wideband thermal noise. Even though Bise reported human responses at similarly low levels, our observation needs independent replication before we would claim it as definitive.

However, the *BioInitiative* report noted “At least five new cell tower studies are reporting bioeffects in the range of 0.003–0.05 μW/cm^**2**^ researchers report headaches, concentration difficulties and behavioral problems in children and adolescents; and sleep disturbances, headaches and concentration problems in adults.” This level corresponds to −36 dBm, an exposure frequently being reported by slow responders in our olmesartan immunostimulation follow-up cohort. After consultation, and some initial data gathering with electromagnetic level meters, we decided to suggest that these slow responders might be wise to take steps to protect themselves from Electrosmog.

### The sleeping caps case series

**Fig. 5:**
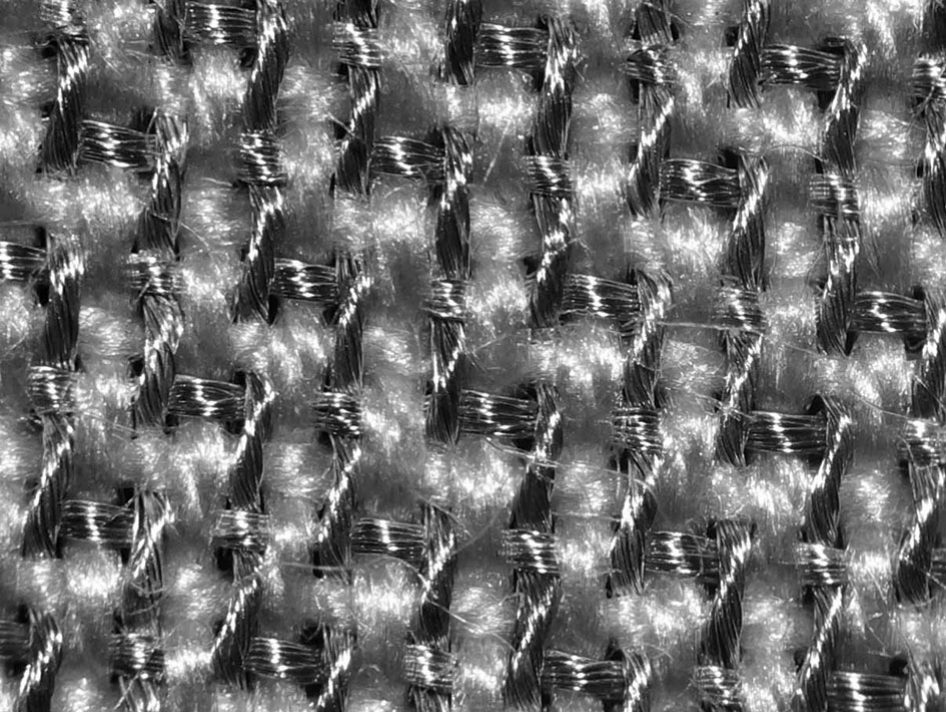
A X20 micrograph of a microwave-blocking fabric woven with a mesh of silver-coated polyester strands among the supporting bamboo fibers

Patients began to initiate protection by purchasing commercially available shielded clothing and tenting from retailers. This clothing typically has silver-coated polyester threads interwoven with the supporting fabric so that the garment is capable of partially blocking microwave Electrosmog (see Fig. 5).

**Fig. 6:**
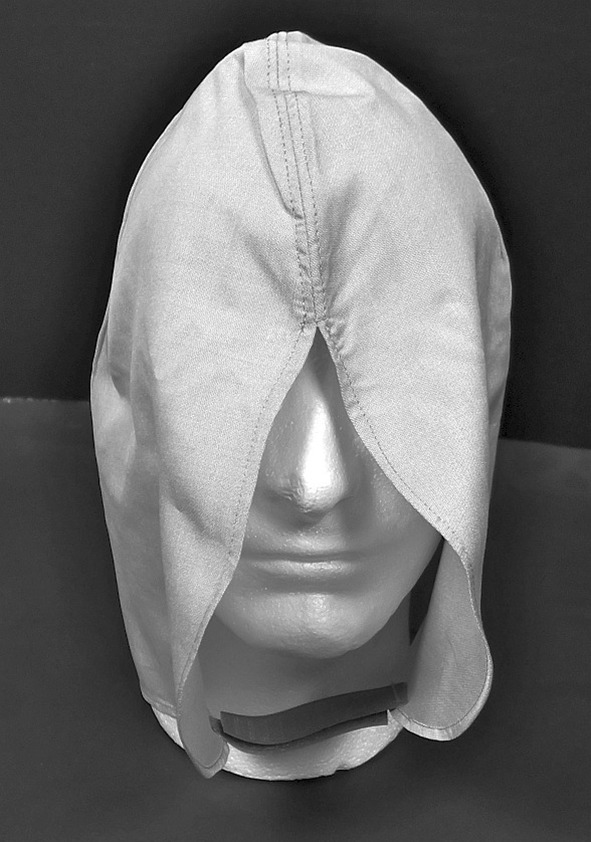
A photograph of a sleeping cap sewn from the microwave-shielding fabric

We quickly realized that frequent anecdotal reports of symptomatic improvement, especially when the brain and brain stem were “shielded” during sleep, warranted standardization with a garment whose electromagnetic shielding performance could be more easily analyzed and optimized. “Sleeping caps” (Fig. 6) were sewn and, upon informed request, distributed free of charge to members of our follow-up olmesartan cohort. A total of 64 patients took part in this case series, with a variety of immune diagnoses including arthritis, lupus, multiple sclerosis, sjogrens and celiac. As these patients were all ill, many undergoing olmesartan treatment with therapeutic intent, we decided that ethical considerations precluded the distribution of “placebo caps” without the silver threads.

**Fig. 7:**
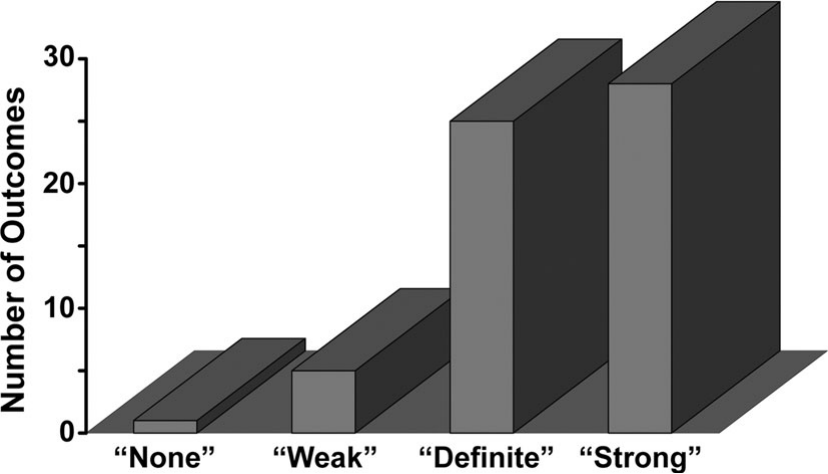
A *bar chart* of the 64 PRO patient responses reporting whether there was no change in symptoms from wearing the cap for 4 h during sleep and work, or a weak, definite, or strong change

We decided to make the reporting task minimally onerous by asking patients to initially wear the cap once for 4 h during sleeping and once for 4 h during normal activity (many are house-bound). We sought patient-reported outcomes (PRO) of whether the garment had “No Effect,” a “Weak Effect,” a “Definite Effect” or a “Strong Effect,” regardless of whether the effect was good or bad (Fig. 7).

A full 90 % of the 64 patients reported a “Definite” or “Strong” change in their symptoms. This compares with the 3 % incidence for electromagnetic hypersensitivity typically expected in the population as a whole [23].

While a placebo or nocebo effect might be expected to bias our PRO data, follow-up reports have indicated a durable response over many months. Additionally, Dieudonne [24] has questioned the likelihood of nocebo causation in EHS.

### Immunopathology from Electrosmog

When the Electrosmog in a patient’s environment is reduced, the immune system tends to become more active. This may result in immunopathology. Indeed, some patients have reported a surge in disease symptoms, occasionally an intolerable surge, after WiFi routers and cell phones have been switched off in their homes. Others have reported that travel to a very quiet area, such as a remote canyon, caused a surge in their immune symptoms.

While further research is needed to clarify these reactions, autoimmune patients seem predisposed to Electrosmog hypersensitivity at levels currently existing in typical home and work environments, and this factor may be affecting their therapeutic response.

## Discussion

The experiments described in this paper confirm that biological molecules are constantly moving and interact with timescales measured in picoseconds. As a result, forces will be exerted on the charged atoms within these molecules by incident electromagnetic fields, including Electrosmog.

There is no reason to suspect that a pulsed electromagnetic wave of 1 μs duration (1000 times slower than a typical molecular response) might cause any less damage to biology than a continuous wave of the same magnitude. It is therefore important to have very-fast-acting peak-reading signal level meters when measuring the biological interaction potential of electromagnetic waves.

Much of the research literature in this field is criticized as not being sufficiently authoritative because experiments have not been conducted under the current pragma of placebo control and simplistic (*p* = 0.05) analysis of results. Research in this area will only move forward when critics start to examine *qualitative* study outcomes—for example, observations which might indicate that a Faraday cage should have been an element of a study’s experimental methodology, or that a 2–3 days acclimatization or immune—washout might have changed the study results.

Furthermore, it seems likely that signals a million times lower than those currently being used in research may be sufficient to elicit a tangible change in human biology. In order to better understand the amplitude at which bioeffects become apparent, it is important that experimental guidelines be delineated which ensure that Electrosmog does not confound a study’s results.

Finally, we need to plan how to handle subjects whose symptoms become untenable (due to immunopathology) during acclimatization to an Electrosmog-quiet environment, or during immune washout. We cannot ignore the increasing body of evidence showing electromagnetic effects on the immune system. The “controversial” nature of electromagnetic hypersensitivity will not diminish until we grasp the complexity of the task we face in defining exactly how electromagnetic waves interact with human biology.

## Electronic supplementary material

Below is the link to the electronic supplementary material.

Supplementary material 1 (MP4 1975 kb)

## Footnotes

1 Our emulation protocol was essentially similar to that of Turton et al. except that we used Gromacs 3.3 running on a multiprocessor Linux operating system. Because the VDR is a much larger molecule than lysozyme, we had to emulate 239 amino acids containing a total of 2415 atoms. They were bathed in thousands of molecules of water. For each of our dozens of experiments, we computed 500,000 emulation steps to get 750 picoseconds of real time data. Each run often took several days of CPU time. We used the NDLP bounding box of Wassenaar [16, 17] to keep the number of water molecule calculations to a minimum.

2 The mathematics package ‘GNU Octave” was used to compute the FFTs in this study. It is an open source mathematics package similar to Wolfram’s MATLAB.

3 Just as there are many units of measurement for ionizing radiation, so there are many ways of measuring non-ionizing electromagnetic radiation. For ionizing, one can choose dose units of Rad, Grey, Roentgen, Rem, Sieverts and the common dps (disintegrations per second, often metered as counts per minute). For non-ionizing, one can use Volts/metre as a measure of field strength, Watts/metre^2^, or Decibels relative to 1 mW (dBm). The authors usually use dBm, as, being logarithmic, the dBm measure can easily denote wide variations in field strengths (it is easier to visualize −7 0dBm than “0.002 V/m”). Once the wave’s frequency is known, it is simple to convert between each non-ionizing unit of measurement.
